# Effect of a Powder Mould in the Post-Process Thermal Treatment of ABS Parts Manufactured with FDM Technology

**DOI:** 10.3390/polym13152422

**Published:** 2021-07-23

**Authors:** Joaquín Lluch-Cerezo, Rut Benavente, María Desamparados Meseguer, Juan Antonio García-Manrique

**Affiliations:** 1 Department of Mechanical Engineering and Materials, Universitat Politècnica de València, Camino de Vera s/n, 46022 Valencia, Spain; rutbmr@upv.es (R.B.); amesegue@mcm.upv.es (M.D.M.); jugarcia@mcm.upv.es (J.A.G.-M.); 2 Engineering Research Team, Florida Universitària, 46470 Catarroja, Spain; 3 Instituto de Tecnología de Materiales, Universitat Politècnica de València, Camino de Vera s/n, 46022 Valencia, Spain

**Keywords:** fused deposition modelling (FDM), post-process thermal treatment, thermal part deformations, acrylonitrile butadiene styrene (ABS), powder mould

## Abstract

The post-process thermal treatment of thermoplastics improves their mechanical properties, but causes deformations in parts, making them unusable. This work proposes a powder mould to prevent dimensional part deformation and studies the influence of line building direction in part deformations in a post-process thermal treatment of 3D printed polymers. Two sets of ABS (acrylonitrile butadiene styrene) test samples manufactured by fused deposition modelling (FDM) in six different raster directions have been treated and evaluated. One set has been packed with a ceramic powder mould during thermal treatment to evaluate deformations and mould effectiveness. Thermogravimetric tests have been carried out on ABS samples, concluding that the thermal treatment of the samples does not cause degradations in the polymeric material. An analysis of variance (ANOVA) was performed to study internal building geometry and mould influence on part deformation after the thermal treatment. It can be concluded that powder mould considerably reduces dimensional deformations during the thermal treatment process, with length being the most affected dimension for deformation. Attending to the length, mould effectiveness is greater than 80% in comparison to non-usage of moulding, reaching 90% when the building lines are in the same direction as the main part.

## 1. Introduction

Fused deposition modelling (FDM) has become the most widely adopted additive manufacturing technology for manufacturing complex structures. This success can mainly be attributed to its extraordinary ability to manufacture complex parts without special tooling, besides significantly reducing material waste, time and cost process of manufacturing prototypes [1]. Despite the proliferation of this manufacturing technology, FDM is used in a few functional parts because they have anisotropic characteristics [2,3,4] and their mechanical properties are usually inferior to those parts fabricated through injection moulding [5]. The mechanical properties of parts manufactured with the FDM process depend on several variables, such as parameters related to process conditions, parts orientation during manufacturing, percentage filling, space between filaments, etc. [6,7,8]. On the other hand, internal micro-void patterns and residual thermal stresses resulting from the manufacturing process also affect the mechanical properties of the final part [9,10,11]. Residual stresses are significant during material consolidation of the deposited layers, due to different factors: thermal or cooling stresses owed to differential cooling and building line orientation, resulting in internal stresses, and quenching stresses caused by cooling too quickly below the glass transition point before thermodynamic equilibrium is archived [12]. Additionally, residual stresses are influenced by the part manufacturing direction and its position during manufacturing [13].

The quality and mechanical properties of FDM parts can be improved if modifications before, on site and after manufacturing are made. In order to improve the quality, some works have developed a control system for the most common additive manufacturing processes and materials, including dimensional parts control [14]. Other studies improve the properties evaluating the effect of infill parameters [15,16], such as layer thickness, raster direction and layup order. These parameters have a significant effect on the mechanical performance. The anisotropic behaviour of parts could be minimized when FDM fabrication process parameters are properly configured [17]. Other authors evaluate the process parameters’ effect on stress accumulation during the deposition process. They conclude that stress accumulations are related to part distortions and depend on building parameters, such as layer thickness and line width [18]. Nevertheless, a proper selection of FDM parameters could improve the mechanical properties and it is possible to reach the obtained result using injection-moulded parts [19].

The most promising results to improve quality and mechanical properties have been found in post-manufacturing processes. One study designed and fabricated a device that uses acetone to modify FDM ABS parts surface to enhanced quality by providing smooth surfaces and fix imperfections [20]. On the other hand, other studies improve dimensional accuracy eliminating edge part defects by the laser-cutting post-process [21]. Thermal annealing is one of the best performing post-processes in FDM. The parts are heated between the glass transition and melting point temperature for a fixed time period and cooled to room temperature [22]. Annealing promotes the relaxation of residual stresses developed during cooling after building process, thanks to the molecular mobility of the amorphous fraction. Residual stresses can be related to dimensional deformations of the part [23]. The literature shows that heating ABS parts above its glass transition temperature produces material reflow that reduces internal micro-voids; therefore, annealing has positive results on the mechanical properties of ABS parts [24]. Annealing post-process increases interlayer adhesion and improves mechanical properties, therefore reducing the internal thermal stress that occurs as a result of manufacturing and the anisotropy of FDM parts. These improvements may significantly modify the structure and properties of FDM parts and depend on annealing temperatures, cooling methods and times, etc. They should be considered by the processing industry [25,26]. However, other studies have shown that thermal post-process annealing affects some infill patterns more than others. Additionally, these thermal treatments can cause deformations in the parts that make them unusable [25,27]. In addition, during the thermal process, mass loss may occur in the parts, which could result in a dimensional decrease of the part. These losses would occur in mass fraction of volatiles [28].

Tensile testing shows more positive results in semi-crystalline materials (PLA and Cu-PLA) than in the amorphous materials (ABS and Al-ASA), since the latter only show a small increment in tensile strength. Nevertheless, under the most aggressive annealing conditions, ABS 3D printed parts’ inter-laminar toughness increases more than 2700% than the non-annealed baseline material [29]. Therefore, if a significant improvement in the properties of ABS parts subjected to thermal post-processing is to be obtained, temperatures and times will have to be increased, leading to unacceptable deformations of the treated parts.

To avoid part deformations during thermal treatments, a recent study has packed complex-shaped parts in sodium chloride powder during the thermal remelting process [30]. An analysis of the treated parts reveals an improved internal structure that increases mechanical and changes optical properties. No noticeable part deformation was observed after the thermal process. However, several samples developed defects due to inadequate powder packing. Another study characterizes deformations and residual stress in FDM ABS parts under thermal treatment, obtaining shrinkages and displacement fields, and relating them to two key processing parameters: raster angle and printing speed [31]. 

Powder packing could be the most promising method to avoid part deformations during the thermal post-process. However, there is currently no work evaluating the effectiveness of this method. This work aims to evaluate the effectiveness of ceramic powder mould packing when aiming to avoid or minimize part deformations in 3D printed parts during thermal post-processes. The temperature and time of the thermal treatment have been increased in comparison with another studies [24] in order to obtain significant part deformations. To avoid undesired reactions during heat treatment, alumina has been selected as ceramic powder, because it is an inert material and does not react with plastic parts.

In this paper, dimensional part deformation in post-process thermal treatment is studied, in addition to the influence of part line building direction on the effectiveness of the mould to avoid part deformations. To reach this objective, variation of part dimensions (length, width and height) will be measured according to their line building direction. The thermal decomposition of the ABS is studied via thermogravimetric analysis (TGA) in order to ensure that mass loss is not noticeable in treated parts and is used to validate dimensional results, because mass loss might affect part dimensions. This work also develops an analysis of variance (ANOVA) to evaluate the effect of internal geometry and mould packaging in part deformation.

## 2. Materials and Methods

### 2.1. Test Specimens Design and Manufacturing

ABS sample geometries manufactured by FDM were printed in the lab and subsequently evaluated. The commercial 2.85 mm White ABS 3D Printer Filament Material (Ultimaker B.V., Gerdelmalsen, The Netherlands) was used. The values of the main thermal properties of ABS material used in this paper are as follows: the melting temperature range was 225–245 °C (test method according to ISO 294-1:2017 [32]) and the glass transition temperature was 97 °C (test method according to ISO 306:2013 [33]). 

**Figure 1:**
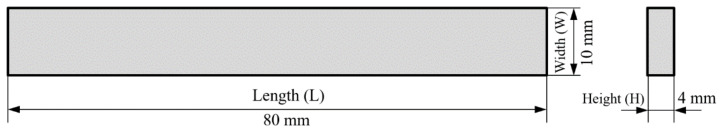
ISO 179-1:2010 Type 1 geometry.

The samples were manufactured according to standard ISO 179-1:2010 [34] Type 1 (Figure 1) due to its external geometrical simplicity, which allowed us to print all building lines in a single direction and avoid stress concentration [16].

**Figure 2:**
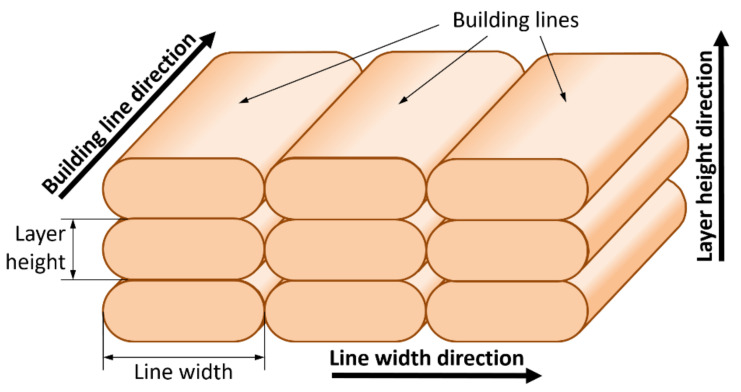
Building line dimensions.

ABS samples were manufactured using an Ultimaker 3 Extended printer (Ultimaker B.V., Gerdelmalsen, The Netherlands) equipped with a 0.4 mm-diameter nozzle. All building lines of each specimen have been printed without differences between infill lines, wall lines and bottom and top layer lines. A unidirectional raster orientation with a 100% density infill line pattern was chosen. In the fabrication process, the following values were used: printing speed of 60 mm/s, temperature of 240 °C and build plate temperature of 80 °C. In order to reduce the interaction between lines of the same layer, a layer height of 0.2 mm and a line width of 0.5 mm were chosen (Figure 2).

Cura software (4.9.1 Version, Ultimaker B.V., Gerdelmalsen, The Netherlands) was used to generate G-code files and to command and control all the process parameters. 3D printed sample geometries were modelled using Inventor software (Version 2019, Autodesk, Inc., San Rafael, CA, USA) and imported to Cura.

#### Internal Geometry

**Figure 3:**
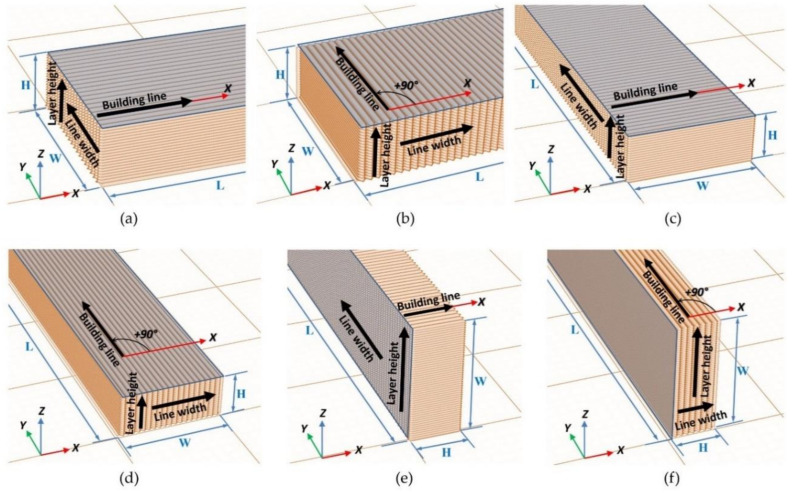
Internal sample geometry code: (**a**) XY + 0; (**b**) XY + 90; (**c**) YX + 0; (**d**) YX + 90; (**e**) YZ + 0; (**f**) YZ + 90.

In order to manufacture and to code samples test, Standard ISO/ASTM 52921:2013 [35] was used to define orthogonal layer orientation with respect to the coordinate system of the 3D printer machine. As the orientation of building lines in each layer is not defined in this Standard, a nomenclature to code each test sample was developed, taking into account the orientation of building direction lines [4]. The samples with six different internal geometries based in building line directions were manufactured and coded as shown in Figure 3.

For each internal geometry, the directions of each dimension of the building lines (Figure 2) can be related to directions of the outer dimensions of the sample: length (L), width (W) and height (H) (Table 1).

### 2.2. Thermal Post-Process Treatment

**Figure 4:**
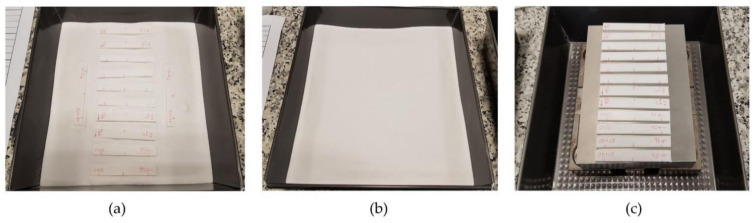
(**a**) Samples set inside of the mould over a powder layer of 1 cm; (**b**) upper alumina powder layer of 1 cm covers samples; (**c**) samples set outside the mould, over the 7 kg metal plate.

The samples set were introduced on a dry alumina powder bed inside a non-stick steel container (Figure 4a) and they were covered with an upper-layer alumina powder (Figure 4b), forming a ceramic mould around specimens in order to avoid deformations due to creep. A one-centimetre-thick alumina powder was used in both layers. Other samples were placed outside the mould (Figure 4c) to compare mould effectiveness. Samples are packed in aluminium oxide powder with granulometry of 150 micrometres and 99.78% purity (Protechno, Girona, Spain). The powder granulometry used was lower than layer height to ensure that no details were lost from the part surface. Ceramic powder was packed around the specimens with a pressure of 12 g/cm^2^, forming an expendable mould. For this purpose, another steel container with a metal plate of 7 kg was placed on top, covering the entire surface of the ceramic powder. To ensure constant pressure in the mould, the metal plate was maintained throughout the thermal process. No binding agents were used because the pressure was enough to ensure that the ceramic powder behaved as a solid.

Samples were heat treated in a convection furnace at a temperature of 135 °C (38 °C above ABS glass transition temperature) for 120 min. A 10 °C/min ramp was used to ensure that temperature inside the mould reached 135 °C. Before unpacking samples, the mould was kept in the furnace until it reached room temperature.

### 2.3. Design of Experiments

The internal geometry and the utilization of ceramic mould for packing samples were the variables considered to study dimensional deformations in samples after the thermal treatment process. The Taguchi’s method for two variables at different levels was used to elaborate the design of experiments (Table 2).

A full factorial design was used to determine the combination of variable levels to use for each experimental case (Table 3). Five replicas of each experiment were carried out.

**Figure 5:**
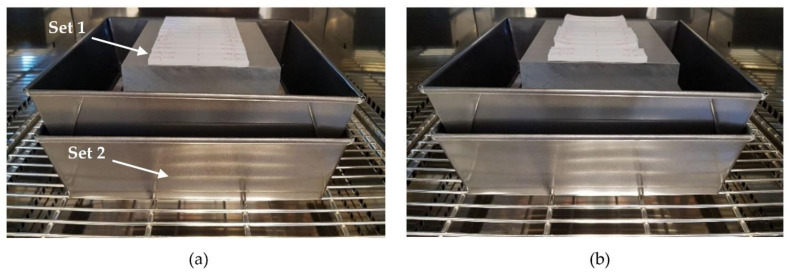
(**a**) Samples before thermal treatment in the furniture; (**b**) after thermal process, deformation is clearly visible in samples of Set 1.

The experimental test was classified in two sets. Set 1 included samples from 1 to 6, where the ceramic mould was not used. Set 2 included samples from 7 to 12, where the ceramic mould was used, allowing us to study the mould effectiveness to prevent dimensional deformation in samples (Figure 5).

**Figure 6:**
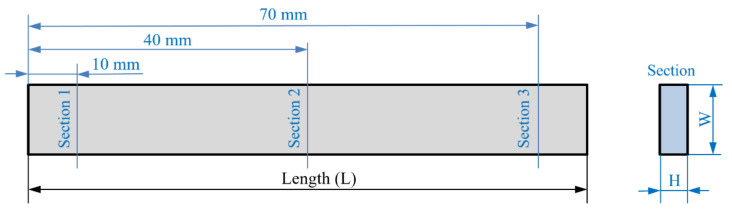
Measured sections in each specimen.

Dimensional measurements (length, width and height) were carried out in all tests to evaluate deformations on samples. The length (L), width (W) and height (H) values of each specimen are taken before and after thermal treatment in order to evaluate dimensional changes. The width (W) and height (H) values were obtained as the average of values in three sections of each specimen (Figure 6). The measurements were carried out with an electronic digital calliper instrument (Resolution = 0.01 mm, Accuracy = ±0.03 mm) according to the standard methods.

In each specimen, ΔL, ΔW, and ΔH are calculated according to ISO 294-4:2018 [36] using Equations (1)–(3). Subscripts _f_ and _o_ indicate, respectively, measurements of each property evaluated after and before the thermal post-process.

(1) $$\Delta L=100\;\cdot \;\frac{L_{f}-L_{o}}{L_{o}}$$

(2) $$\Delta W=100\;\cdot \;\frac{W_{f}-W_{o}}{W_{o}}$$

(3) $$\Delta H=100\;\cdot \;\frac{H_{f}-H_{o}}{H_{o}}$$

### 2.4. Thermogravimetric Analysis

The thermal decomposition of the samples was carried out with a Thermogravimetric Analyzer (TGA-Q50, TA Instruments, NewCastle DE, USA). A Ni standard reference was used for the temperature calibration of TGA. The nitrogen flow was 40.0 mL min^−1^ through the balance. Samples were spread on a platinum pan and the gas over the sample was nitrogen or air at 60.0 mL min^−1^.

## 3. Results and Discussion

Table 4 shows the average measurements observed in variations of length (ΔL), width (ΔW) and height (ΔH) for all specimens in each sample. Negative values represent a decrease in percentage, while positive values represent an increase in percentage in every evaluated dimension.

**Figure 7:**
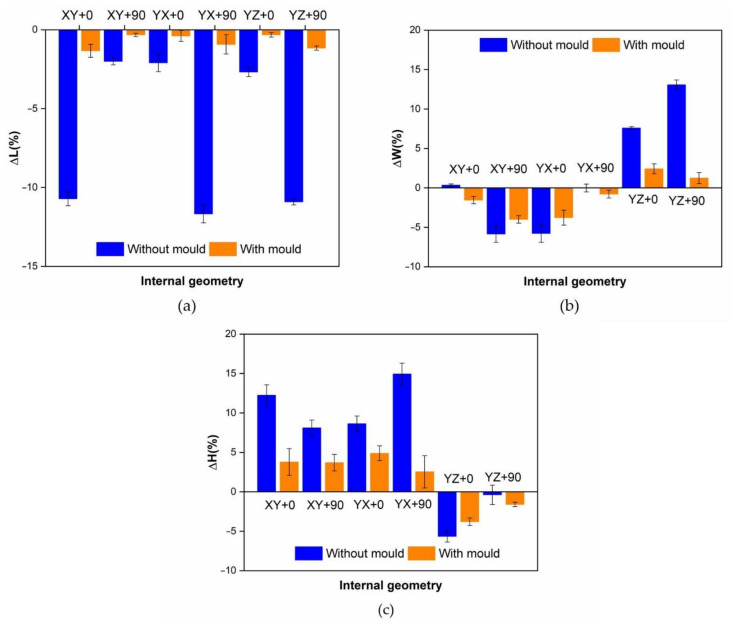
Variations attending to internal geometry and powder mould usage. (**a**) Length variations; (**b**) width variations; (**c**) height variations.

The values obtained for the average dimensional variations are represented in the bar charts in Figure 7. It can be observed that using a ceramic powder mould reduces the dimensional variations in the samples after thermal treatment, while the dimensional variation in samples without the mould during thermal treatment is quite significant.

The results show a decrease in the length (L) and the width (W), and an increase in the height (H) dimension for XY + 0, XY + 90, YX + 0 and YX + 90 internal geometries. On the other hand, in YZ + 0 and YZ + 90 internal geometries, a decrease in length (L) and height (H), and increase in width (W) dimension is observed. Shrinkage is always higher in the direction of the building lines, and the mould reduces deformations in all cases.

An analysis of variance (ANOVA) was performed to quantify the mould effectiveness to avoid dimensional variations during the thermal process in samples with different building directions (Table 5, Table 6 and Table 7). In order to analyse the significant effect of the factors on responses, an F test with a level significance of 0.05 has been used. Table 5, Table 6 and Table 7 show that internal geometry and the use of ceramic mould have significant effect on all dimensional changes produced after thermal post-process.

### 3.1. Internal Geometry Influence

Fisher’s Least Significant Difference (LSD) procedure was used to discriminate between mean values. No statistically significant differences were observed between the internal geometry XY + 0 vs. YX + 90 and XY + 90 vs. YX + 0 in any of the dimensions analysed (Table 8). These pairs of internal geometries have been included because some studies [13] show that in some 3D printers the position of parts in the build plate can generate residual tensions and subsequent deformations. However, in these tests, the 3D printer has not generated differences between them.

As shown by the ANOVA and bar charts (Figure 7), a strong influence of internal geometry on the dimensional variations of samples was observed. Samples made with building lines in the main sample direction (XY + 0, YX + 90 and YZ + 90 samples) show five times more length deformation than the samples made with building lines in a perpendicular direction to the main sample direction (XY + 90, YX + 0 and YZ + 0). If a mould is used, three times longer deformation is shown. Attending to width (W) deformation, samples with building lines in the direction of the width of the part (XY + 90 and YX + 0) present elevated shrinkage. In samples with building lines in the height direction (YZ + 0 and YZ + 90), shrinkage only appears in this direction. It can be concluded that there is a relationship between building line directions and the shrinkages in the part.

Table 9 shows deformations in the building line, line width and layer height directions. The deformation of samples during the thermal treatment mainly appears in the direction of the building lines and layer height. Elongations always appear in the layer height direction, because building lines shrink the part in its direction. There is a major deformation in the layer height direction than in the line width direction due to the larger contact area between building lines in this direction (Figure 2). It demonstrates the influence of these parameters in part deformations during the thermal process. On the other hand, the average deformations in the line width were ten times smaller than in the layer height direction due to the small contact zone, because the value of the line width is 0.5 mm, instead of the 0.2 mm value of the layer height. If a mould is used, this difference decreases up to five times.

In Table 9, samples can be grouped in two sets: XY + 0, YX + 90, YZ + 90 in the first set and XY + 90, YX + 0, YZ + 90 in the second one. Deformations in building line, line width and layer height directions are similar in every set. Deformations during the thermal treatment are more important if the building line direction matches the main length of the part. All these effects are observed independently if a mould is used or not. For this reason, in order to reduce deformations, building lines directions at 90° to the longest dimension of the part should be selected to manufacture it. 

### 3.2. Ceramic Mould Effectiveness

Using a ceramic mould during the thermal post-process has a clear influence on decreasing all dimension deformations (Figure 7). Nevertheless, the length (Figure 7a) is the most affected dimension for deformation. For this reason, length will be used to evaluate mould effectiveness in all internal geometries studied.

In order to measure mould effectiveness, Equation (4) is used, E being the mould effectiveness to avoid deformations in the length L during the thermal post-process.

(4) $$E=1-\frac{\Delta L_{\mathrm{with}\;\mathrm{mould}}}{\Delta L_{\mathrm{without}\;\mathrm{mould}}}$$

The mould effectiveness for each evaluated direction is shown in Table 10.

It can be observed that mould effectiveness decreased when parts are fabricated with XY + 90 y YX + 0 internal geometries, due to the fact that the largest shrinkages during the thermal process were produced in the direction of the manufacturing lines.

**Figure 8:**
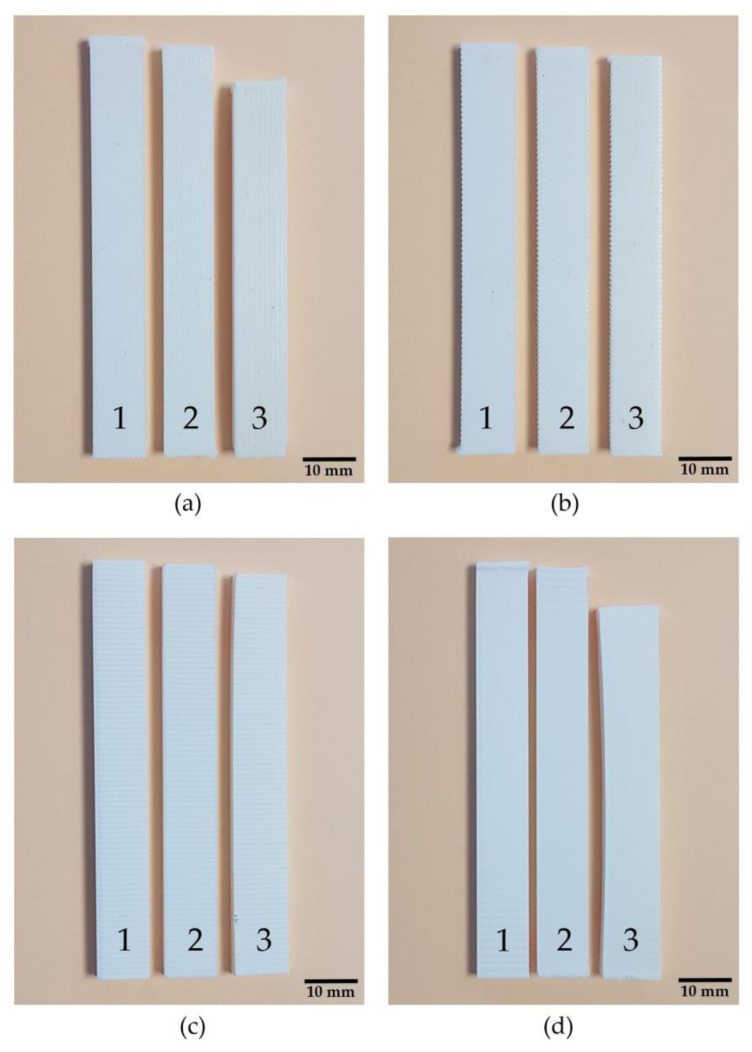
Specimen comparison before thermal process (1), after thermal process with mould (2) and after thermal process without mould (3). (**a**) XY + 0 Samples; (**b**) XY+90 Samples; (**c**) YZ + 0 Samples; (**d**) YZ + 90 Samples.

Deformations in significant internal geometries are shown in Figure 8. In all cases, the effectiveness of the mould is clearly visible because it reduces sample deformation. Nevertheless, samples with internal geometries XY + 0 and YZ + 0 are less affected by deformations in a thermal post-process. It can be concluded that mould is less necessary when building lines are perpendicular to main part dimension. On the other hand, XY + 0 and YZ + 90 samples are stronger affected by deformations, being the mould that is highly recommended when the building lines are in the same direction that main part dimension.

Figure 8 shows that, in order to avoid deformations in a thermal post-process without using a mould, the best strategy is to use XY + 90 or YZ + 90 internal geometries, as observed in Table 4.

### 3.3. Thermogravimetric Analysis

In order to determine whether the thermal treatments performed on ABS samples modify their composition, thermogravimetric tests have been carried out [28,37].

**Figure 9:**
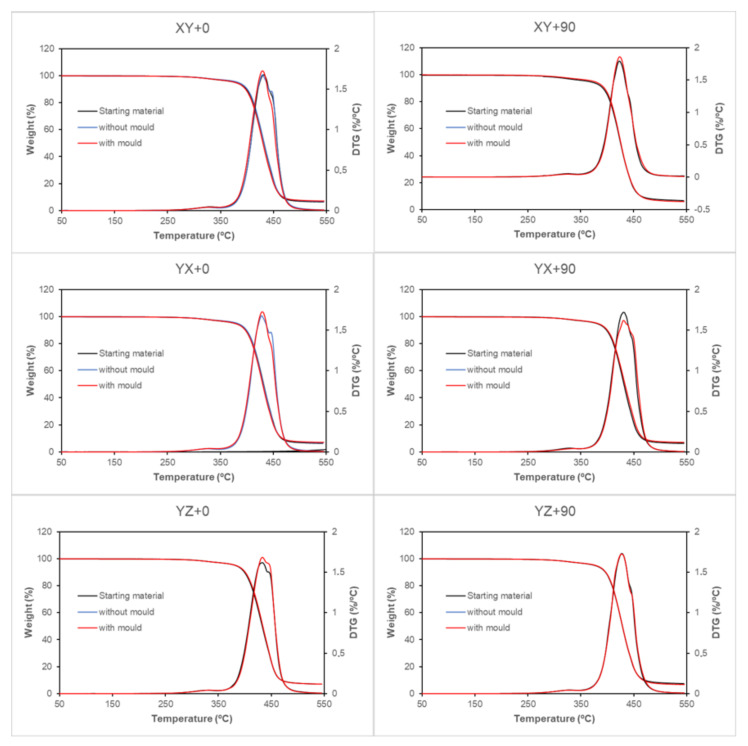
TG/DTG curves of ABS samples at 10 °C min^−1^ in nitrogen atmosphere. Specimens comparison before thermal process, after thermal process with mould and after thermal process without mould for XY + 0, XY + 90, YX + 0, YX + 90, YZ + 0 and YZ + 90 samples.

Figure 9 shows the thermogravimetric/differential thermogravimetric (TG/DTG) curves before thermal process, after thermal process with mould and after thermal process without mould for XY + 0, XY + 90, YX + 0, YX + 90, YZ + 0 and YZ + 90 for ABS samples.

A small degradation step was observed between room temperature and 350 °C with mass loss less than 4 %. The main degradation step starts and ends between 350 and 550 °C. The residue at 550 °C is in the vicinity of 7 %. From an inspection of the DTG curves, it is clear that the main degradation of ABS takes place in two steps. The first degradation step corresponds to the loss of the volatile compounds [37,38]. The second step corresponds to the breaking of the stronger bonds from the reticulated product of the first step, referring mainly to the scission of C-C main chain bonds [38,39].

In Figure 9, it can be observed that the two degradation steps are consecutive and appear partially overlapping in the range 350–550 °C; in this research, to simplify the calculations, the separate steps will not be considered. Thus, two temperature ranges have been established for weight loss measurements: from 25 to 350 °C (light fraction) and from 350 to 550 °C (heavy fraction). The inorganic residue was obtained at 550 °C.

The weight loss and the residue obtained for the different samples tested as a function of the heat treatment followed are shown in Table 11.

In all cases, the thermal degradation of ABS presents the same profile, there being no significant differences in the degradation percentages of the samples before heat treatment and after heat treatment with and without mould. In weight loss 1, it can be seen that the thermal treatment slightly reduces the fraction, although this reduction is less than 0.3%. Weight loss 2, with values around 90%, sees its values modified by less than 1.5%. The residue fraction modifies its values around 0.6%. These slight differences in the fractions are not related to the geometry with which the specimens are manufactured, nor to the use of the mould in the thermal process, which indicates that the thermal treatment carried out on the specimens does not cause degradations in the polymeric material.

## 4. Conclusions

Post-process thermal treatment has positive results on the mechanical properties of thermoplastics. However, thermal treatment can cause deformations in the parts that make them unusable. Annealing treatments produce a relaxation of the samples. When working with semi-crystalline materials, manufacturing process as injection or extrusion, with rapid cooling, causes highly amorphous structures, depending on the direction of the fibres in the extrusion process and their length. Annealing gives the polymer the energy necessary to recrystallize, although long production times are required.

This paper evaluates the use of a ceramic powder mould during thermal post-process of ABS 3D printed parts in order to decrease dimensional variations in specimens. The influence of internal geometry direction with the building line, line width and layer height directions on specimen deformations caused by thermal treatment is also studied.

When an ABS sample obtained by extrusion is subjected to an annealing process, deformations occur due to the movement of polymeric chains towards a more ordered microstructure. This deformation can be controlled, even prevented, if a mould is used during the thermal cycle. The ceramic power mould minimizes the dimensional changes of the sample in all the internal geometries studied, although the influence of the building line direction on the final deformation is observed.

ABS samples are manufactured according to standard ISO 179-1:2010 Type 1. Thermogravimetric tests have been carried out to determine if ABS-sampled deformations could be associated with evaporation of any volatile species, concluding that the thermal treatment carried out on the specimens does not cause degradations in the polymeric material.

An analysis of variance (ANOVA) was performed to study mould influence and internal geometry of the part in deformation during the annealing treatment. Using a ceramic powder mould considerably reduces dimensional deformations that occur during the annealing process. Length is the most affected dimension due to deformation of the sample. For this reason, length has been used to evaluate mould effectiveness. This effectiveness is greater than 80% in all internal geometries evaluated, in comparison to non-usage of mould, reaching 90% when the building lines are in the same direction as the main part.

After evaluating the annealed specimens, it can be concluded that building line directions at 90° to the longest dimension of the part have a major influence on part deformation. On the other hand, the use of a ceramic powder mould considerably reduces dimensional deformations that occur during the annealing process. Specimens building with XY + 0 and YZ + 90 internal geometries are stronger affected by deformations, making it the most highly recommend powder mould when the building lines are in the same direction as the main part.

## Figures and Tables

**Table 1:** Relationship between dimensions of the building lines and the directions of the sample dimensions for each type of internal sample geometry.

Internal Sample Geometry	Building Line Direction	Line Width Direction	Layer Height Direction
XY + 0	L	W	H
XY + 90	W	L	H
YX + 0	W	L	H
YX + 90	L	W	H
YZ + 0	H	L	W
YZ + 90	L	H	W

**Table 2:** Variables and levels.

Level	1	2	3	4	5	6
Internal geometry	XY + 0	XY + 90	YX + 0	YX + 90	YZ + 0	YZ + 90
Ceramic mould	NO	YES				

**Table 3:** Experimental parameters.

Test	Internal Geometry	Ceramic Mould
1	XY + 0	NO
2	XY + 90	NO
3	YX + 0	NO
4	YX + 90	NO
5	YZ + 0	NO
6	YZ + 90	NO
7	XY + 0	YES
8	XY + 90	YES
9	YX + 0	YES
10	YX + 90	YES
11	YZ + 0	YES
12	YZ + 90	YES

**Table 4:** Average dimensional changes after thermal treatment.

		Set 1 (without Mould)				Set 2 (with Mould)		
Internal Geometry	Test	ΔL (%)	ΔW (%)	ΔH (%)	Test	ΔL (%)	ΔW (%)	ΔH (%)
XY + 0	1	−10.72	0.34	12.25	7	−1.34	−1.56	3.77
XY + 90	2	−2.01	−5.85	8.10	8	−0.33	−4.00	3.68
YX + 0	3	−2.09	−5.78	8.63	9	−0.39	−3.78	4.88
YX + 90	4	−11.68	−0.02	14.94	10	−0.93	−0.79	2.53
YZ + 0	5	−2.68	7.60	−5.66	11	−0.33	2.42	−3.80
YZ + 90	6	−10.91	13.08	−0.39	12	−1.16	1.24	−1.61

**Table 5:** ANOVA for ΔL.

Source	Sum of Squares	Df	Mean Square	F Ratio	*p* Value
A: Internal geometry	0.0349	5	0.0070	487.32	0.0000
B: Mould	0.0528	1	0.0528	3686.22	0.0000
INTERACTIONS					
AB	0.0246	5	0.0049	343.45	0.0000
RESIDUALS	0.00069	48	0.000014		
TOTAL (CORRECTED)	0.1130	59			

**Table 6:** ANOVA for ΔW.

Source	Sum of Squares	Df	Mean Square	F Ratio	*p* Value
A: Internal geometry	0.1236	5	0.0247	535.59	0.0000
B: Mould	0.0105	1	0.0105	226.51	0.0000
INTERACTIONS					
AB	0.0342	5	0.0068	148.37	0.0000
RESIDUALS	0.0022	48	0.000046		
TOTAL (CORRECTED)	0.1705	59			

**Table 7:** ANOVA for ΔH.

Source	Sum of Squares	Df	Mean Square	F Ratio	*p* Value
A: Internal geometry	0.1508	5	0.0302	212.69	0.0000
B: Mould	0.0336	1	0.0336	237.17	0.0000
INTERACTIONS					
AB	0.0324	5	0.0065	45.77	0.0000
RESIDUALS	0.0068	48	0.00014		
TOTAL (CORRECTED)	0.2237	59			

**Table 8:** Dimension deformation showing significant differences between internal geometries (* denotes a statistically significant difference).

Contrast	Sig. L	Difference L	Sig.W	Difference W	Sig. H	Difference H
XY + 0 vs. XY + 90	*	−0.04861	*	0.04318	*	0.02121
XY + 0 vs. YX + 0	*	−0.04783	*	0.04173	*	0.01253
XY + 0 vs. YX + 90		0.00276		−0.00201		−0.00724
XY + 0 vs. YZ + 0	*	−0.04524	*	−0.05614	*	0.12738
XY + 0 vs. YZ + 90		0.00007	*	−0.07763	*	0.09010
XY + 90 vs. YX + 0		0.00078		−0.00145		−0.00868
XY + 90 vs. YX + 90	*	0.05137	*	−0.04519	*	−0.02845
XY + 90 vs. YZ + 0		0.00337	*	−0.09932	*	0.10617
XY + 90 vs. YZ + 90	*	0.04868	*	−0.12081	*	0.06889
YX + 0 vs. YX + 90	*	0.05059	*	−0.04374	*	−0.01977
YX + 0 vs. YZ + 0		0.00259	*	−0.09787	*	0.11485
YX + 0 vs. YZ + 90	*	0.04790	*	−0.11936	*	0.07757
YX + 90 vs. YZ + 0	*	−0.04800	*	−0.05413	*	0.13462
YX + 90 vs. YZ + 90		−0.00269	*	−0.07562	*	0.09734
YZ + 0 vs. YZ + 90	*	0.04531	*	−0.02149	*	−0.03728

**Table 9:** Deformations between dimensions of the building lines and the directions of the sample dimensions for each type of internal sample geometry.

	Set 1 (without Mould)			Set 2 (with Mould)		
Internal Geometry	Building Line Direction (%)	Line Width Direction (%)	Layer Height Direction (%)	Building Line Direction (%)	Line Width Direction (%)	Layer Height Direction (%)
XY + 0	−10.72	0.34	12.25	−1.34	−1.56	3.77
XY + 90	−5.85	−2.01	8.10	−4.00	−0.33	3.68
YX + 0	−5.78	−2.09	8.63	−3.78	−0.39	4.88
YX + 90	−11.68	−0.02	14.94	−0.93	−0.79	2.53
YZ + 0	−5.66	−2.68	7.60	−3.80	−0.33	2.42
YZ + 90	−10.91	−0.39	13.08	−1.16	−1.61	1.24

**Table 10:** Mould effectiveness.

Internal Geometry	Mould Effectiveness E (%)
XY + 0	87.52
XY + 90	83.64
YX + 0	81.19
YX + 90	92.03
YZ + 0	87.75
YZ + 90	89.34

**Table 11:** Weight loss and residue obtained.

	Starting Material				Set 1 (without Mould)				Set 2 (with Mould)		
Internal Geometry	Weight Loss 1 (%)	Weight Loss 2 (%)	Residue (%)	Test	Weight Loss 1 (%)	Weight Loss 2 (%)	Residue (%)	Test	Weight Loss 1 (%)	Weight Loss 2 (%)	Residue (%)
XY + 0	3.2	90.1	6.7	1	3.1	90.2	6.9	7	3.2	89.7	7.2
XY + 90	3.6	89.0	6.9	2	3.4	90.1	6.5	8	3.4	90.3	6.3
YX + 0	3.2	90.1	6.7	3	2.9	90.2	6.9	9	3.2	89.6	7.2
YX + 90	3.1	90.3	6.6	4	2.9	89.8	7.2	10	2.9	90.0	7.1
YZ + 0	3.1	89.8	7.4	5	2.9	90.1	6.9	11	2.9	90.0	7.1
YZ + 90	3.2	89.3	7.5	6	3.2	90.8	5.9	12	3.1	90.0	6.9
